# Skin TLR7 Triggering Promotes Accumulation of Respiratory Dendritic Cells and Natural Killer Cells

**DOI:** 10.1371/journal.pone.0043320

**Published:** 2012-08-22

**Authors:** Holger Hackstein, Nicole Hagel, Angela Knoche, Sabine Kranz, Jürgen Lohmeyer, Werner von Wulffen, Olivia Kershaw, Achim D. Gruber, Gregor Bein, Nelli Baal

**Affiliations:** 1 Institute for Clinical Immunology and Transfusion Medicine, Justus-Liebig-University Giessen, Member of the German Center for Lung Research, Giessen, Germany; 2 Department of Internal Medicine II, Giessen, Germany; 3 Department of Internal Medicine, V Pulmonary Diseases and Comprehensive Pneumology Center, Klinikum Grosshadern, Ludwig-Maximilians University, Munich, Germany; 4 Department of Veterinary Pathology, Freie Universität Berlin, Berlin, Germany; Oklahoma Medical Research Foundation, United States of America

## Abstract

The TLR7 agonist imiquimod has been used successfully as adjuvant for skin treatment of virus-associated warts and basal cell carcinoma. The effects of skin TLR7 triggering on respiratory leukocyte populations are unknown. In a placebo-controlled experimental animal study we have used multicolour flow cytometry to systematically analyze the modulation of respiratory leukocyte subsets after skin administration of imiquimod. Compared to placebo, skin administration of imiquimod significantly increased respiratory dendritic cells (DC) and natural killer cells, whereas total respiratory leukocyte, alveolar macrophages, classical CD4+ T helper and CD8+ T killer cell numbers were not or only moderately affected. DC subpopulation analyses revealed that elevation of respiratory DC was caused by an increase of respiratory monocytic DC and CD11b^hi^ DC subsets. Lymphocyte subpopulation analyses indicated a marked elevation of respiratory natural killer cells and a significant reduction of B lymphocytes. Analysis of cytokine responses of respiratory leukocytes after stimulation with Klebsiella pneumonia indicated reduced IFN-γ and TNF-α expression and increased IL-10 and IL-12p70 production after 7 day low dose skin TLR7 triggering. Additionally, respiratory NK cytotoxic activity was increased after 7d skin TLR7 triggering. In contrast, lung histology and bronchoalveolar cell counts were not affected suggesting that skin TLR7 stimulation modulated respiratory leukocyte composition without inducing overt pulmonary inflammation. These data suggest the possibility to modulate respiratory leukocyte composition and respiratory cytokine responses against pathogens like Klebsiella pneumonia through skin administration of a clinically approved TLR7 ligand. Skin administration of synthetic TLR7 ligands may represent a novel, noninvasive means to modulate respiratory immunity.

## Introduction

Imiquimod is a synthetic Toll-like receptor TLR7 ligand stimulating innate and adaptive immunity against viruses and tumors [1], [2]. Clinically, imiquimod crème is a FDA-approved immune response modifier that is indicated for the skin treatment of actinic keratosis, superficial basal cell carcinoma and external genital warts [3], [4]. Plasmacytoid and myeloid dendritic cells as well as B cells are the major leukocyte subpopulations expressing TLR7 [5], [6]. TLR7 activation of these professional antigen presenting cells bridges innate and adaptive immunity through induction of inflammatory cytokines type I interferon, TNF-α, IL-12, chemokines and DC migration [7]–[9]. As a consequence, TLR7 triggering through imidazoquinolines, such as imiquimod [10], results in enhanced T helper type 1 (Th1) immune responses and reduced Th2 immune responses. Interestingly, different groups demonstrated effectiveness of TLR7 triggering to suppress allergic airway inflammation in experimental asthma models suggesting immunomodulatory potential of synthetic TLR7 ligands [11]–[13]. Low-dose TLR7 stimulation has been reported to inhibit TLR9-induced IFN-α production in human and mouse plasmacytoid DC revealing immunoregulatory potential of TLR7 ligands [14]. Accordingly, experimental skin vaccination studies with imiquimod revealed significant co-induction of T regulatory cells and IL-10 signalling suppressing the activity of cytotoxic CD8+ T cells [15].

However, systemic administration of TLR7 ligands has been associated with chronic immune activation, inflammation and lymphoid disruption resembling HIV-mediated pathology as well as systemic endothelial activation raising significant concerns with respect to drug toxicity and safety [16], [17]. Accordingly, systemic administration of the related imidazoquinoline resiquimod in chronic HCV-infected patients has been associated with frequent severe grade adverse events including systemic cytokine induction, fever, shivering and lymphopenia [18].

Recently, Pasmatzi et al reported that skin administration of imiquimod induced significant alterations in peripheral blood lymphocytes of healthy individuals indicating the possibility that skin TLR7 triggering may represent a novel, less toxic strategy to modulate systemic immunity [19]. Moreover, repeated skin administration of imiquimod in mice has been reported to significantly increase the survival of mice bearing intracranial glioma and breast cancer suggesting that skin triggering of TLR7 may result in systemic immunmodulation [20]. Skin administration of imiquimod (Aldara® creme) as a single agent in tumor-bearing mice was associated with intracranial elevation of tumor infiltrating CD4+ T helper and CD8+ T killer lymphocytes [20]. These data suggested the possibility that skin administration of imiquimod can modulate leukocyte populations in other organs.

In this placebo-controlled study we have analysed for the first time the hypothesis that skin administration of a synthetic TLR7 ligand may be capable of modulating the composition of respiratory leukocyte subsets. By means of multiparameter flow cytometry we have investigated alterations of all major leukocyte subsets, including dendritic cell (DC) subpopulations (plasmacytoid DC, monocytic DC, CD103+ DC, CD11b^high^ DC) and innate lymphocyte subsets (natural killer cells, gamma delta TCR positive lymphocytes). We have included lung DC subsets into the analysis because they play critical roles as instigators and modulators of immunity [21], [22]. Our results revealed that skin administration of a synthetic TLR7 ligand applied to the back skin promoted significant alterations of respiratory monocytic DC, CD11b^high^ DC, NK cells and B cells. Notably, in contrast to modulating respiratory DC, NK and B cell numbers, skin administration of imiquimod did not result in overt pulmonary inflammation as indicated by normal lung histology and normal cell counts in bronchoalveolar lavage.

## Results

### Skin TLR7 Stimulation Induces Accumulation of Respiratory DC in Contrast to Macrophages and Granulocytes

**Figure 1:**
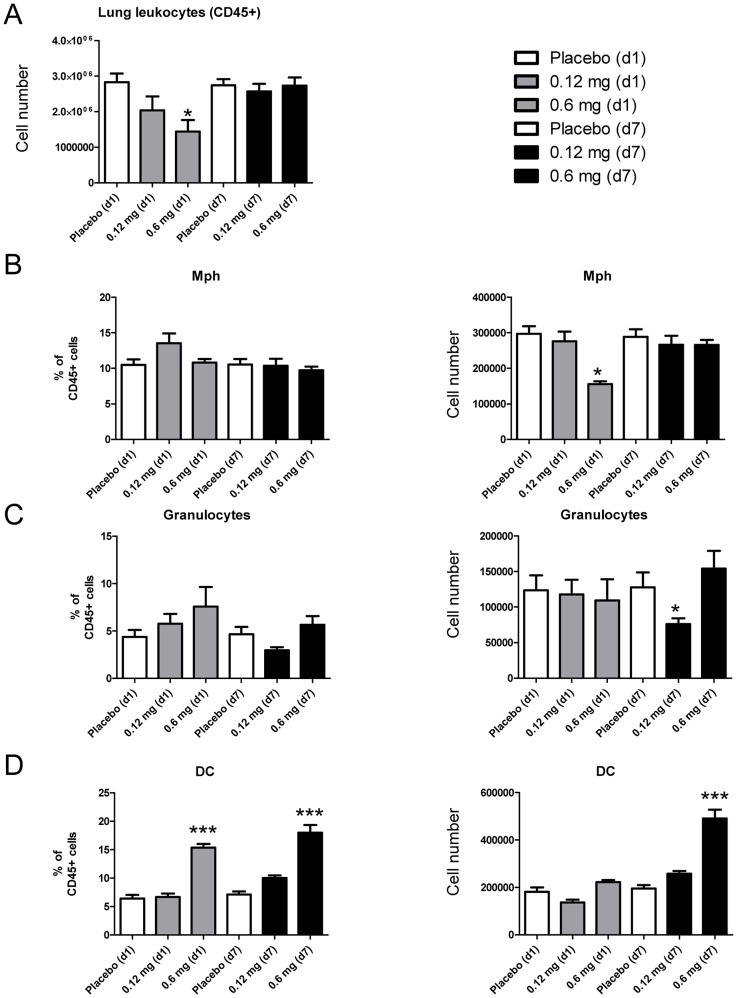
Skin application of imiquimod increases relative and absolute respiratory DC numbers. Skin of mice was treated with TLR7 ligand imiquimod or placebo in a daily fashion and relative and absolute respiratory leukocyte subsets were quantitated by flow cytometry at the indicated time points (see gating stategy in Fig. S1). Total lung leukocytes (A), respiratory macrophages (B), respiratory granulocytes (C) and respiratory DC numbers after imiquimod exposure (D). Mean ± SEM; n ≥4; *p<0.05; **p<0.01; ***p<0.001 versus placebo.

To assess the effects of skin TLR7 triggering on respiratory leukocytes, mice were treated with imiquimod creme (Aldara®) applied to the back skin for up to seven days. To control for unspecific effects related to drug formulation or treatment procedure, control animals were treated with placebo crème consisting of identical ingredients except for imiquimod. Sustained TLR7 skin triggering over 7 days did not affect total respiratory leukocyte numbers, whereas single exposure resulted in reduced leukocyte numbers (Fig. 1A).

Analysis of leukocyte populations indicated normal or reduced absolute numbers of macrophages (CD45+ SiglecF+ F4/80+; Fig. 1B) and respiratory granulocytes (CD45+; F4/80^low^/^neg^; CD11b^++^, GR-1^++^; Fig. 1C). In contrast, both relative and absolute respiratory DC (CD45+, CD11c+, SiglecF^neg^, NK1.1^neg^) numbers were elevated after sustained skin TLR7 triggering with imiquimod over 7 days (0.6 mg imiquimod; p<0.001; Fig. 1D). The employed gating strategy facilitated the distinction of these subsets (Fig. S1). Within the CD11c+ cells, autofluorescent CD11c^+^ alveolar macrophages were identified based on SiglecF expression and excluded from the DC analysis (Fig. S1). Absolute respiratory DC numbers increased 151% after 7 days when compared to placebo-treated control animals (491322±36872 versus 195059±15390; Fig. 1D; p<0.001) suggesting a marked effect of skin TLR7 triggering on respiratory DC. Accordingly, the DC/Macrophage ratio in lung tissue markedly increased after 7d skin application of 0.6 mg imiquimod from 0.7 (±0.08) to 1.9 (±0.2; p<0.001).

### Monocytic DC and CD11b^high^ DC Represented the Major Respiratory DC Subsets Increased after Skin TLR7 Triggering

**Figure 2:**
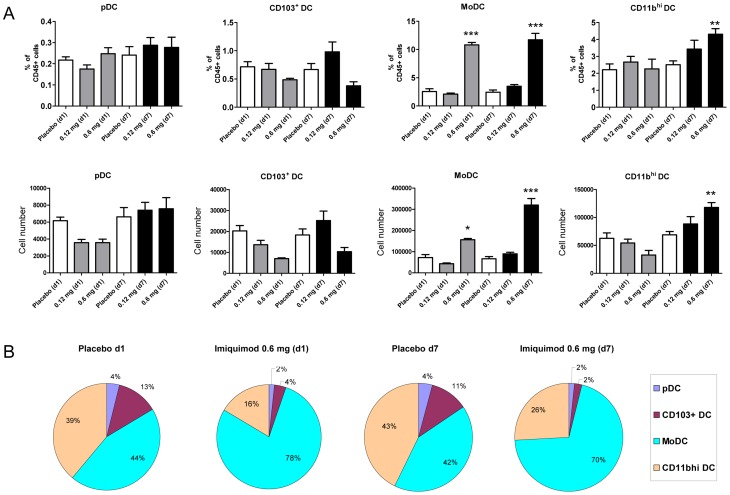
Accumulation of respiratory DC after skin TLR7 triggering is caused by an increase of monocytic DC and CD11b^high^ DC subsets. Modulation of respiratory DC subsets pDC, CD103^+^ DC, monocytic DC and CD11b^high^ DC (see gating strategy in Fig. S2) after skin imiquimod exposure versus placebo as described in materials and methods (A, B). Mean ± SEM; n ≥4; *p<0.05; **p<0.01; ***p<0.001 versus placebo.

In order to determine which specific DC subset is elevated after skin TLR7 administration we performed 8-colour flow cytometry and quantitated plasmacytoid DC, CD103^+^ DC, monocytic DC and CD11b^high^ DC. Dissection of the four major DC subsets was performed based on expression of 120g8, CD103, CD11b and MHC-class II (I-A^b^; Fig. S2). DC subset analysis revealed that monocytic DC represented the principle DC subpopulation increased after topical TLR7 triggering (Fig. 2A). Relative monocytic DC frequencies increased from 2–3% of all CD45+ respiratory leukocytes to ≥10% in 0.6 mg imiquimod-treated mice (p<0.001; Fig. 2A). Interestingly, significant elevation of respiratory monocytic DC was detectable already after 24 h (p<0.001) suggesting that a single imiquimod dose is sufficient to elevate respiratory monocytic DC (Fig. 2A). Additionally, relative and absolute CD11b^high^ DC numbers were also elevated after sustained skin TLR7 application over 7 days (Fig. 2A). In contrast, pDC and CD103^+^ DC were not or only moderately affected. Given the fact that skin TLR7 triggering changed both absolute DC numbers and relative DC subsets in the lung we next questioned how imiquimod affected the composition of the DC compartment in the lung. In comparison to placebo-treated mice, imiquimod significantly increased the monocytic DC frequency among all respiratory DC from 42–44% up to 70–78% (Fig; 2B p<0.001). Interestingly, modulation of respiratory DC composition was detectable within 24 h. Relative DC composition of d1 and d7 placebo-exposed mice were almost identical indicating that DC composition was not affected by the placebo treatment (Fig. 2B).

### Skin TLR7 Triggering Modulates Expression of Costimulatory and Inhibitory Molecules on Respiratory DC

**Figure 3:**
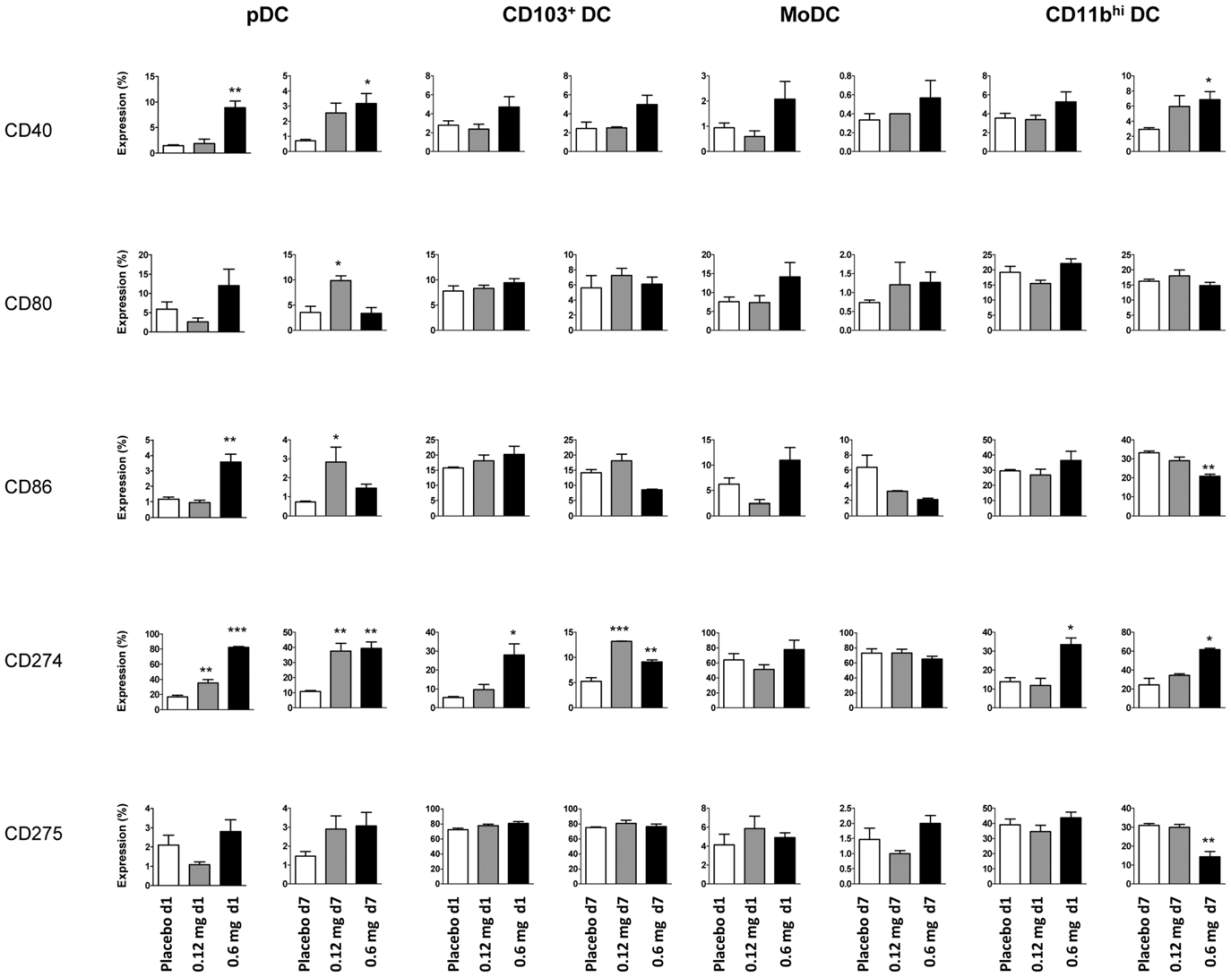
Expression of costimulatory and regulatory surface moelcules on respiratory DC subsets after skin TLR7 triggering. Surface expression of CD40, CD80, CD86, CD274 and CD275 on respiratory DC subsets after 1d and 7d skin imiquimod exposure versus placebo. Respiratory DC subsets were gated as described in Fig. S2. Mean expression ± SEM; n ≥3; *p<0.05; **p<0.01; ***p<0.001 versus placebo.

Analysis of costimulatory molecule (CD40, CD80, CD86) and regulatory B7 family member (CD274, CD275) expression indicated that skin TLR7 triggering affected surface expression of respiratory DC. On pDC, CD40, CD86 and CD274 expression was increased in comparison to placebo controls (Fig. 3). Interestingly, expression of the regulatory B7 family member CD274 was markedly increased on pDC, CD103^+^ DC and CD11b^high^ DC after skin TLR7 triggering (Fig. 3).

### Reduction of Respiratory B Lymphocytes and Elevation of Respiratory Natural Killer Cell Numbers after Skin Imiquimod Exposure

**Figure 4:**
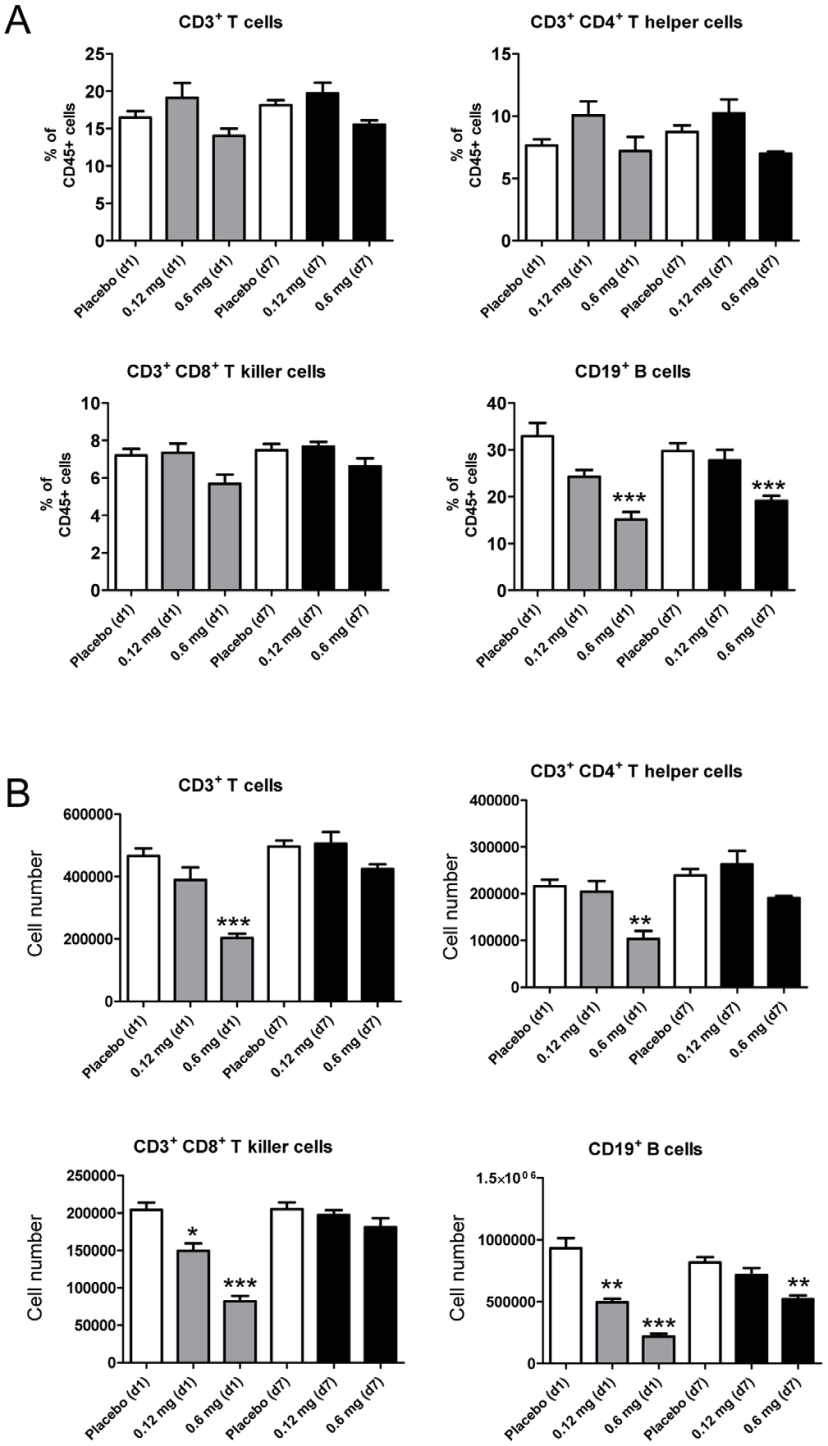
Respiratory T and B lymphocyte numbers are suppressed after skin TLR7 stimulaton. Relative (A) and absolute (B) respiratory T and B lymphocyte subsets were quantitated by flow cytometry (see gating strategy Fig. S3) after skin imiquimod treatement. Mean ± SEM; n ≥4; *p<0.05; **p<0.01; ***p<0.001 versus placebo.

**Figure 5:**
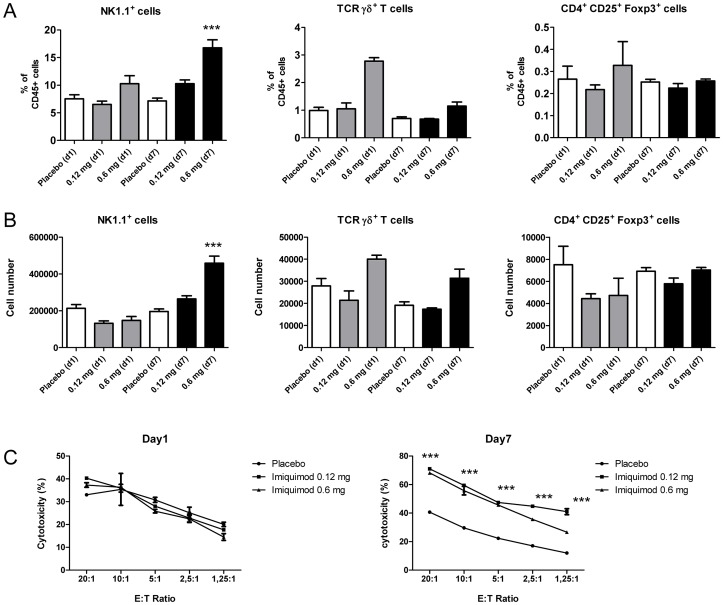
Respiratory NK cell numbers are increased after skin TLR7 triggering. Relative (A) and absolute (B) respiratory NK cells, γδ T lymphocytes and CD4^+^CD25^+^Foxp3^+^T regulatory cells were quantitated by flow cytometry (see gating strategy Fig. S3) after skin imiquimod treatement. Cytotoxicity (C) of equal numbers of magnetic bead enriched respiratory NK cells were determined against Yac-1 target cells after 1d and 7d imiquimod exposure. Mean ± SEM; n ≥4; *p<0.05; **p<0.01; ***p<0.001 versus placebo.

Classical T and B lymphocyte populations, NK cells and γδ TCR+ T cells were gated according to conserved surface markers whereas T regulatory CD4+ T cells were identified after intracellular FoxP3 staining (Fig. S3). Analysis of lymphocyte subpopulations revealed a temporary reduction of absolute CD3+ T cells including CD4+ T helper and CD8+ T killer cells after 1d imiquimod exposure, whereas respiratory CD3+ T cell numbers were not affected after 7d imiquimod exposure (Fig. 4A, B). In contrast, relative and absolute CD19+ B cell numbers were significantly reduced both after 1d and 7d drug exposure suggesting a marked effect of skin imiquimod on respiratory B lymphocytes (Fig. 4A, B). In contrast, sustained skin imiquimod exposure over 7 days significantly increased relative and absolute numbers of respiratory NK cells (Fig. 5A, B) suggesting an effect of imiquimod on innate lymphocytes. After 7 days of skin TLR7 triggering, absolute numbers of respiratory NK cells increased from 195856 (±14504) to 458312 (±39096) per lung preparation (p<0.001; Fig. 5B). Interestingly, functional analysis of cytotoxic activity of equal numbers of magnetic bead-enriched respiratory NK cells indicated increased cytotoxic activity of 7d imiquimod exposed animals suggesting that sustained skin TLR7 triggering may promote respiratory NK activity (Fig. 5C). Skin imiquimod application affected also respiratory γδ TCR+ CD3 T cells and to a lesser extent CD4^+^CD25^+^Foxp3^+^ T regulatory cells, but these differences were statistically not significant (Fig. 5A, B).

### Topical TLR7 Triggering Affects Respiratory Cytokine Production

**Figure 6:**
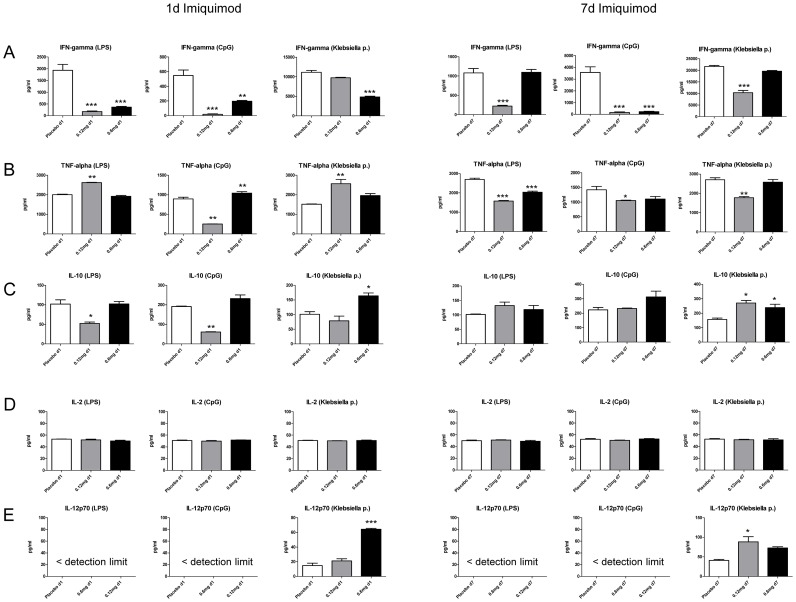
Skin TLR7 triggering modulates respiratory cytokine production. Respiratory leukocytes were CD45^+^ microbead purified from 1d and 7d imiquimod or placebo-treated animals and IFN-γ (A), TNF-α (B), IL-10 (C), IL-2 (D), IL-12p70 (E) and IL-23 were quantitated after LPS, CpG or Klebsiella pneumonia lysate stimulation. IL-23 was below the detection limit (27 pg/ml) in all groups. In unstimulated cells, cytokines were not detectable (detection limit 27 pg/ml). Mean ± SEM; n ≥3; *p<0.05; **p<0.01; ***p<0.001 versus placebo.

Having established that skin TLR7 triggering can modulate respiratory leukocyte numbers we questioned whether cytokine production (IFN-γ; IL-2; IL-10, IL-12p70, IL-23, TNF-α) was also affected. Accordingly, equal numbers of CD45+ microbead-purified respiratory leukocytes were stimulated via TLR4 with LPS and via TLR9 with CpG oligonucleotides. Moreover, to analyse the cytokine response against a typical respiratory pathogen, leukocytes were also stimulated with Klebsiella pneumonia lysate. Results revealed that IFN-γ was significantly reduced in d1 and d7 animals after LPS, CpG and Klebsiella lysate stimulation (Fig. 6). With respect to TNF-α and IL-10, d7 animals showed a trend towards decreased TNF-α and increased IL-10 production after low dose imiquimod exposure in contrast to d1 animals (Fig. 6). IL-23 was below the detection limit and IL-2 production did not differ between the groups. IL-12p70 was only detectable after Klebsiella pneumonia stimulation and exhibited elevated levels in imiquimod-exposed animals. In summary the results indicate the possibility that skin TLR7 triggering may promote a respiratory cytokine response being characterized as decreased IFN-γ, decreased TNF-α, increased IL-10 and increased IL-12p70.

### Modulation of Respiratory Leukocyte Numbers after Skin TLR7 Triggering is Associated with Peripheral Blood Changes

**Figure 7:**
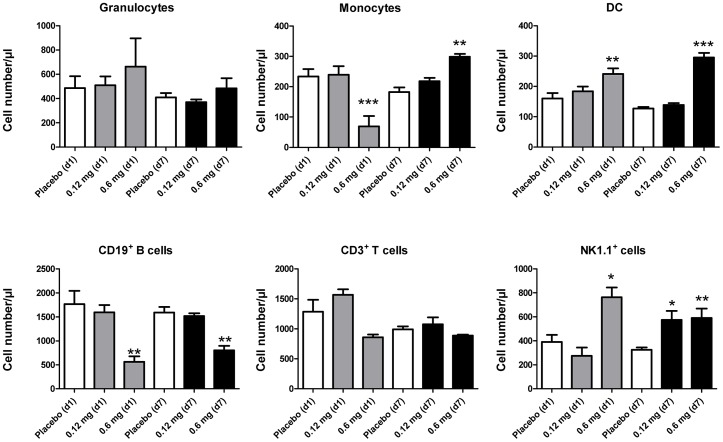
Respiratory leukocyte subset changes are associated with modulation of corresponding peripheral blood leukocyte subsets. Peripheral blood leukocyte subsets were quantitated by flow cytometry after skin imiquimod exposure. Mean/ul ± SEM; n ≥3; *p<0.05; **p<0.01; ***p<0.001 versus placebo.

Since respiratory leukocytes are repopulated through the peripheral blood we analyzed changes in major blood leukocyte subsets after skin TLR7 administration. Results indicated that peripheral blood DC, B lymphocytes and natural killer cells exhibited similar changes after skin TLR7 administration suggesting a direct association of peripheral blood changes with respiratory leukocyte subsets after skin imiquimod exposure (Fig. 7).

### Topical TLR7 Triggering does not Induce a Bronchoalveolar Inflammatory Response

**Figure 8:**
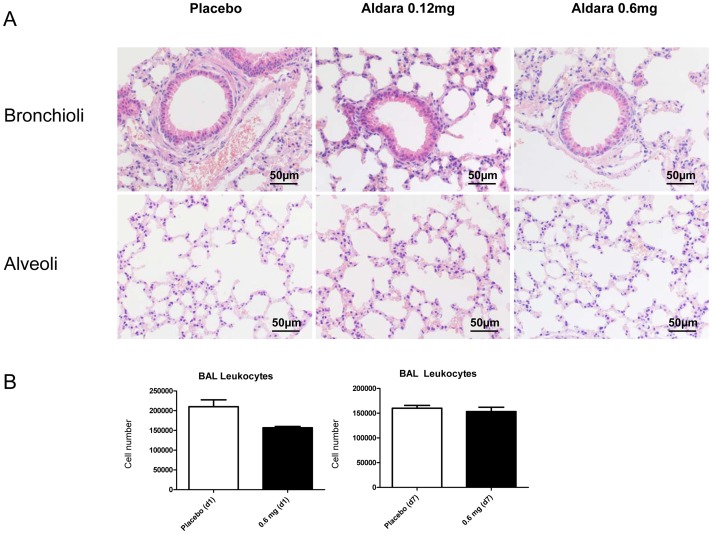
Skin TLR7 stimulation does not induce lung histopathology or alveolar leukocytosis. Lung sections (A) and BAL leukocyte numbers (B) of mice exposed to imiquimod or placebo. (A) shows histopathology of d7 animals. Histopathology of d1 animals exhibited similar results (Fig. S5). BAL leukocyte numbers from d1 and d7 treated animals after imiquimod exposure (B). Mean ± SEM; n ≥3.

The marked modulation of respiratory leukocyte composition raised the question whether sustained skin TLR7 triggering has induced signs of pulmonary inflammation. Accordingly, lungs of sacrificed animals were analysed in a blinded manner by pathology and leukocyte numbers after bronchoalveolar lavage were quantitated. Lung histology indicated no signs of pathology; in fact lungs from imiquimod exposed d1 and d7 animals were not different from placebo-exposed animals (Fig. 8A; Fig. S5). Accordingly, leukocyte numbers after bronchoalveolar lavage were not affected in comparison to placebo-exposed controls (Fig. 8B). These results are in agreement with the total respiratory leukocyte numbers in lung homogenate (Fig. 1A) and indicate that skin TLR7 ligand administration was not inducing pulmonary inflammation.

### Topical TLR7 Triggering Modulates Skin Dendritic Cell CD86 and CD274 Expression

**Figure 9:**
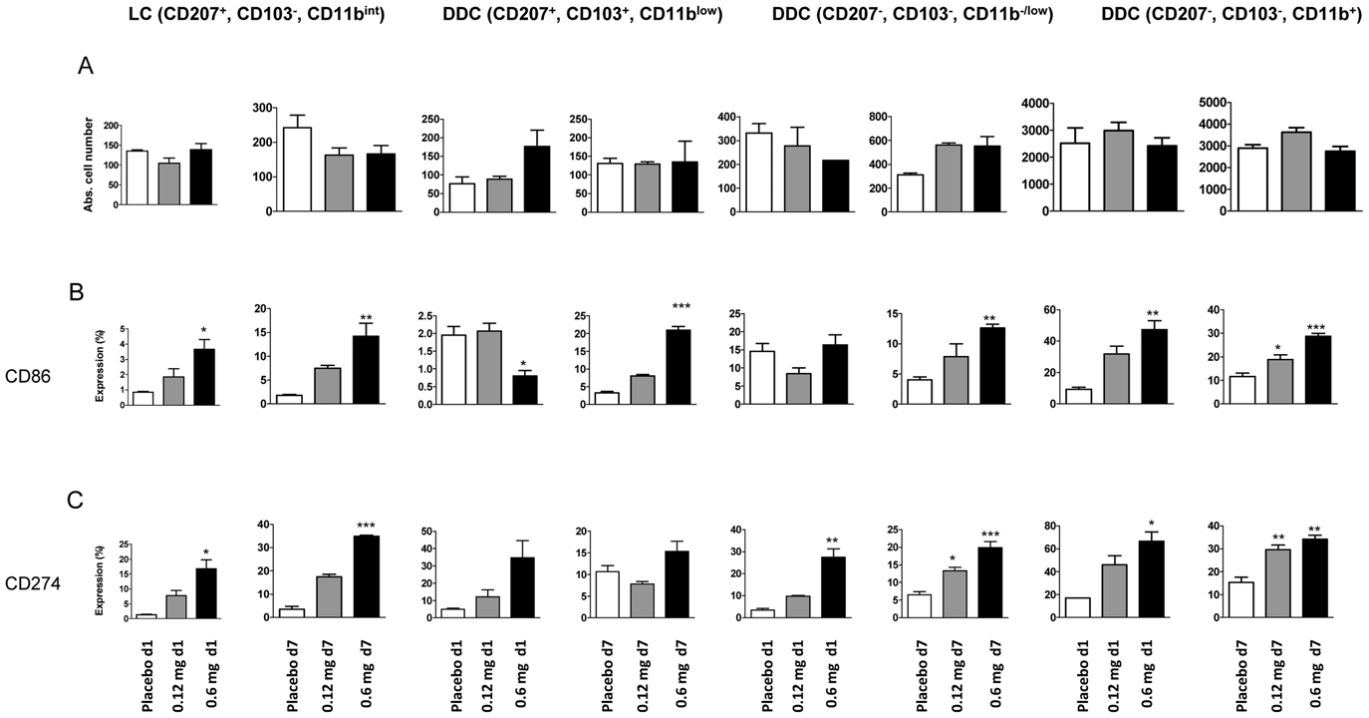
Topical TLR7 triggering affects skin dendritic cell CD86 and CD274 expression. Absolute skin DC subset numbers from 2cm^2^ skin (A) and subset specific expression of CD86 (B) and CD274 (C) were determined by flow cytometry after 1d and 7d imiquimod exposure as described in the Materials and Methods. Among the CD11c^+^ MHC-class II^+^ CD45^+^ skin DC, Langerhans cells (LC) were identified based on CD207^+^, CD103^-^, CD11b^int^ expression profile. Different dermal DC (DDC) subsets were discriminated based on heterogenous expression of CD207, CD103, CD11b. Mean ± SEM; n ≥2-3. *p<0.05; **p<0.01; ***p<0.001 versus placebo.

CD11c+ MHC-class II+ skin DC subsets were discriminated into Langerhans cells and dermal dendritic cell subsets according to CD207 (langerin), CD103 and CD11b expression (Fig. S4). Results indicated no significant changes in absolute DC subset numbers after skin imiquimod exposure. However, in contrast, analysis of DC maturation status indicated significant upregulation of CD86 and CD274 surface expression in most skin DC subsets, already after 1d skin imiquimod exposure (Fig. 9). These results suggested that topical skin TLR7 triggering affected maturation of skin DC.

## Discussion

Here we presented the results of a placebo-controlled animal study investigating the modulation of respiratory leukocyte subset numbers after skin administration of the TLR7 ligand imiquimod. Results revealed the possibility to modulate respiratory leukocyte composition after skin TLR7 triggering without inducing pulmonary inflammation as indicated by normal histopathology and BAL counts. Skin TLR7 triggering markedly elevated absolute and relative respiratory DC and respiratory natural killer cell numbers and reduced respiratory B cell numbers. Notably, most of the changes were already detectable within 24 h and sustained skin TLR7 triggering over 7 days further enhanced the effects. The results are specifically related to TLR7 triggering since control animals receiving placebo crème exhibited no changes. Additionally, skin TLR7 triggering also modulated cytokine production of respiratory leukocytes as indicated by reduced TNF-α and increased IL-10 production.

So far, the effects of TLR7 stimulation have been primarily studied with respect to peripheral blood and lymphoid tissue changes but the modulation of *respiratory* leukocytes after *skin* TLR7 triggering has been unknown. Gunzer and colleagues reported that a single systemic dose of the TLR7 ligand R848 resulted in a transient depletion of peripheral blood leukocytes due to increased endothelial adhesiveness [16]. In agreement, we also observed a reduction of peripheral blood B lymphocytes, T lymphocytes and monocytes after one day skin imiquimod exposure.

However, the smaller leukocyte subsets of NK cells and DC were increased already after one day imiquimod exposure. Sustained systemic exposure to TLR7 ligands over 7 days has been reported to induce a chronic inflammatory condition in the murine lymphoid system and disruption of the lymphoid structure [17]. Accordingly, we have analyzed the histology of lung tissue sections after 7 day skin TLR7 agonist exposure and did not find any signs of pulmonary inflammation. The absence of lung pathology after 7 day imiquimod exposure, associated with normal total respiratory leukocyte and BAL numbers suggested that skin imiquimod application did not induce a chronic respiratory inflammation. Rather, the results indicated that skin imiquimod application modulated the composition of respiratory leukocytes as well as the cytokine producing capacity. Interstingly, recently it was reported that intranasal administration of the related TLR7 ligand resiquimod did also induce transient accumulation of respiratory NK, DC and granulocytes [13]. Our results extend these findings and suggest that skin TLR7 ligand administration is able to modulate the leukocyte composition of the lungs.

It can be argued that the applied imiquimod doses in the discussed experimental studies are higher than those used in the clinical situation. However, recently it was reported that sustained skin application of imiquimod to humans significantly modulated peripheral blood lymphocyte numbers when compared to sex-and age-matched controls receiving vehicle [19]. These results support the hypothesis that skin TLR7 ligand application principally is able to modulate systemic leukocyte numbers. Additionally, repeated skin administration of imiquimod in mice has been reported to significantly improve the survival of animals bearing intracranial tumors [20] supporting the concept of systemic immunomodulation after skin TLR7 triggering. In agreement with this concept, we did find significant modulation of respiratory leukocyte cytokine production capacity even at low imiquimod doses (0.12 mg/d) that did exert no or only minor effects on respiratory leukocyte numbers. Respiratory leukocytes of animals receiving 0.12 mg imiquimod for seven days produced significantly less TNF-α and IFN-γ, both after LPS, CpG and Klebsiella pneumonia lysate stimulation when compared to placebo-exposed controls. These results suggested that modulation of leukocyte cytokine production capacity can be detected at lower doses than modulation of leukocyte numbers after skin TLR7 ligand application. Interestingly, Klebsiella pneumonia lysate stimulated respiratory leukocytes of animals receiving low dose skin TLR7 triggering over 7 days produced significantly higher amounts of immunosuppressive IL-10 and IL-12p70. Moreover, analysis of respiratory DC surface markers and respiratory NK cell cytotoxic activity suggested that skin imiquimod exposure modulated additional functional variables of respiratory leukocytes.

These results suggested the possibility that sustained skin TLR7 triggering may promote respiratory immunoregulation. In agreement with this hypothesis, it was shown that topical imiquimod therapy in a murine breast cancer model was associated with high IL-10 levels and IL-10 blockade markedly improved antitumor activity of imiquimod [23].

In summary, the presented placebo-controlled animal study showed that skin TLR7 triggering with imiquimod modulated respiratory DC and NK cell numbers as well as respiratory cytokine production capacity. Modulation of respiratory leukocytes was not associated with signs of pulmonary inflammation indicating the possibility that skin TLR7 triggering may represent a novel non-invasive means to modulate respiratory immune responses. Although this study was not designed to investigate the molecular mechanism of elevation of respiratory DC and NK cells, the results reveal novel insight into the in vivo activity of imiquimod. Additional studies are needed to dissect the molecular pathways involved in respiratory immunomodulation after skin TLR7 triggering.

## Materials and Methods

### Ethics, Mice and Treatment

Animal experiments were performed after approval of the ethics authority board Regierungspräsidium Gießen Hessen (file #29/2009). Specific-pathogen-free C57BL/6 (C57BL/6NCrl) mice, 10–12 weeks of age were purchased from Charles River, Sulzfeld, Germany and maintained under specific-pathogen-free conditions. 0.12–0.6 mg/d imiquimod (Aldara® 5% crème; Meda Pharma, Germany) were applied for 1–7 days to the shaved back skin of sedated animals. Shaving of a 2 cm^2^ back neck skin area per mice was done after anesthesia with xylazoline hydrochloride (Bayer, Germany). Back neck skin was selected as application area, because it prevented licking off the drug by the animals. Imiquimod (0.12 mg, 0.6 mg) was applied with a pipet directly onto the shaved skin and it was waited 1–2 min until the drug was absorbed into the skin. In long term treated animals, repeated applications were done after isofluorane anesthesia. Control animals were treated in a manner identical except they received placebo creme (provided by Meda Pharma) instead of imiquimod creme. After drug or placebo application, animals were separated into single cages until the end of the experiment.

### Lung Preparation and Histology

Lung single cell suspension were prepared after enzymatic digestion as described in detail [24] with minor modifications. Briefly, mice were euthanized and lungs were perfused via the right ventricle with HBSS (PAA, Germany) to remove the intravascular pool of cells. Tissues were minced and digestion was performed in 0.09 U/ml type A collagenase (Roche, Germany) and 9.09 U/ml DNase (Roche, Germany) in IMDM (PAA, Germany) with 10% FCS (PAA, Germany) at 37°C for 1h. Single cell suspension were prepared by tissue resuspension with 20 G 1 ½ canules (0.9×40 mm; BD, Germany) and by mashing through a 70 µM cell strainer (BD, Germany). Red blood cells were lysed by ammoniumchloride lysis. Cells were washed with HBSS for flow cytometry staining or leukocytes were magnetic-bead sorted after washing with PBS/2% BSA/2 mM EDTA (PAA, Germany). Bronchoalveolar lavages (BAL) were performed as described [25]. Lung (tissue) samples were fixed in 10% formalin and routinely embedded in paraffin. Sections were cut at 5 µm thickness, dewaxed and stained with hematoxylin and eosin (H&E).

### Flow Cytometry

Cellular phenotyping was performed on a FACS CantoII flow cytometer (Becton Dickinson, San Jose, CA, USA) as described [26] with minor modifications. The following fluorochrome-labelled monoclonal antibodies conjugated to FITC, PE, PE-TxR, PeCy7, PerCPCy5.5, APC, APC-Cy7, Pacific Blue and appropriate isotype controls were used for surface staining according to the manufacturer’s instructions: CD3e, CD4, CD8a, CD11b, CD11c, CD19, CD25, CD45, NK1.1, CD90, CD103, TCR-γδ, I-A^b^, GR1, F4/80, CD40, CD80, CD86, CD274, CD275 (all mabs from Biolegend, Germany), Siglec-F (BD Biosciences, Germany), Langerin (CD207; 929F301, Imgenex, SanDiego, USA) and 120g8 (Dendritics, France). Intracellular Foxp3 staining was performed with the Foxp3 staining set (eBiosciences, Germany) according to the manufacturer instructions. Absolute leukocyte numbers were determined by using trucount beads (Becton Dickinson, Germany) according to the manufacturer instructions.

### Skin Cell Preparation and DC Analysis

Skin cells were prepared as described [27] with minor modifications. Briefly, pieces of skin were removed and subcutaneous fat was removed with forceps. Skin pieces were digested for 45 min at 37 C with 3 ml digestion cocktail. Digestion cocktails contains collagenase XI (4129 U/ml; Sigma, Germany, #C9407-100 mg); hyaluronidase (260 U/ml; Sigma, #H3506-100 mg) and DNase (0.1 mg/ml, Roche) diluted in RPMI 1640 with L-glutamine (PAA Laboratories), penicillin/streptomycin (PAN Biotech) and 10% heat inactivated FCS (PAA Laboratories). Digested skin was minced and washed twice with ice-cold medium with 10 mM EDTA and filtered through a 70 µM cell strainer (BD, Germany). Skin DC were identified by flow cytometry after gating for CD45^+^; MHC-class II^+^ (I-A^b^) and CD11c^+^ cells. Subsequently, Langerhans cells (LC) and dermal dendritic cells (DDC) subsets were dissected based on CD207, CD11b and CD103 expression according to Henri et al. [28].

### Cytokine Quantification

2×10^5^ respiratory leukocytes were bead-purified with CD45 microbeads (Miltenyi Biotec, Germany) according to the manufacturer instruction. Cells were stimulated in 96-well plates (Greiner, Germany) with LPS (1 µg/ml; 0111:B4 strain, Sigma Aldrich, Germany), or CpG ODN 1826 (Invivogen, France) for 24 h in RPMI 1640 medium supplemented with L-glutamine, penicillin/streptomycin, 10% heat-inactivated FCS (PAA Germany). Klebsiella pneumonia lysate (Serotype 2, ATCC 43816) was prepared after overnight suspension culture. Bacteria were concentrated after centrifugation (15 min, 1000 g), diluted in 5 ml 0.9% NaCl, heated for 15 min.at 65°C in a water bath and used at 10 µl/ml for stimulation experiments. Mouse IFN-γ, TNF-α, IL-2, IL-10, IL-12p70 and IL-23 were quantitated by cytometric bead array (flow cytomix, eBioscience, Germany).

### Natural Killer Cytotoxicity Assay

Flow cytometry cytotoxicity assay was performed according to the principles described by Kane et al. [29]. Respiratory effector NK cells were enriched using CD49b microbeads according to manufacturer instructions (Miltenyi Biotec, Bergisch-Gladbach, Germany). Effector cells were incubated at the indicated effector/target (E/T) ratios in culture medium with carboxyfluorescein diacetate succinimidyl ester (CFDA-SE) labeled Yac-1 target cells (Cell line Service, Eppelheim, Germany; 2×10^4^/well) in 96 well round bottom wells (Greiner) for 8 h. Culture medium consisted of RPMI 1640 with L-glutamine (PAA Laboratories), penicillin/streptomycin (PAN Biotech) and 10% heat inactivated FCS (PAA Laboratories). Molar CFDA-SE labeling concentration of target cells was 1 µm and was performed according to the manufacturer instructions (Vybrant CFDA-Cell Tracer kit, Molecular probes, Eugene, USA). Dead cells were quantitated by flow cytometry after adding Sytox Blue (Invitrogen, Germany). Percent lysis was calculated as follow: Percent lysis = Percent dead Yac-1 cells: (CFDA+ Sytox+ double positive cells/CFDA+ cells)×100. Spontaneous lysis was determined in control YAC-1 cultures without NK effectors and was substracted from the actual lysis. Spontaneous lysis was consistently lower than 5%.

### Statistical Analyses

Data are shown as means and SEM. Statistical analyses were performed with Prism 5.02 software (Graphpad software, Inc.). The significance of differences between groups were analysed by one-way ANOVA with Tukey’s post-hoc -test for multiple comparisons. A p-value <0.05 was considered significant.

## Supporting Information

Figure S1 **Flow-cytometry based identification of respiratory macrophages, granulocytes and DC (A-C).** Leukocytes in lung cell suspensions were identified as CD45^+^ cells. Among the CD45^+^ respiratory leukocytes, macrophages were identified based on Siglec-F and F4/80 expression (A). Respiratory granulocytes were identified as CD45^+^ cells, F4/80^low-neg^ and CD11b^++^ GR-1^++^ (B). Respiratory DC were identified within the CD45^+^ cells after gating first for the CD11c^+^ Siglec-F^neg^ cells to exclude highly autofluorescent alveolar macrophages. Subsequently, among the remaining cells, NK1.1^++^ NK cells were gated out to precisely identify respiratory DC (C). Representative gating of n>10 experiments.
(TIF)

Figure S2 **Gating strategy for discrimination of respiratory DC subsets. Respiratory DC were further dissected based on 120g8; CD103, CD11b and I-A^b^ (MHC-class II) expression.** Among the respiratory DC fraction, plasmacytoid DC were identified based on 120g8^+^ CD11b^neg^ expression. Out of the non-plasmacytoid DC fraction; CD103^+^ DC were identified based on CD103 and MHC-class II expression. The remaining respiratory DC were examined for CD11b and MHC-class II (I-A^b^) expression dividing them into two major groups: monocytic DC (CD11b^++^, MHC-class II^low^, CD103^neg^, 120g8^neg^ NK1.1^low^, Siglec F^neg^, CD11c+; CD45+) and CD11b^hi^ DC (CD11b^++^, MHC-II^high^, CD103^neg^, 120g8^neg^ NK1.1^low^, Siglec F^neg^, CD11c+; CD45+). Representative gating of n>10 experiments.
(TIF)

Figure S3 **Gating strategy for discrimination of respiratory lymphocyte subpopulations (A) and regulatory T cells (B).** First, classical CD3+ T cells and CD19+ B cells were identified within the CD45^+^ SSC^low^ respiratory leukocytes, Out of the CD3+ T cell fraction, respiratory T helper and T killer cells were identified based on CD4 and CD8 expression, respectively (A). Additionally, among the CD45^+^ SSC^low^ fraction, respiratory NK cells were identified as CD3^neg^ NK1.1^+^ cells and γδ T cells were identified as CD3^+^ γδ TCR^+^ cells (A). Respiratory T regulatory cells were identified within the CD45^+^ SSC^low^ fraction as CD4+CD25+ cells and then examined for Foxp3 expression (B). Foxp3 gates were set according to control staining with identical mabs except for Foxp3 isotype-matched control (so-called fluorescence minus 1 control; B). Representative gating of n>10 experiments.
(TIF)

Figure S4 **Gating strategy for discrimination of skin DC subpopulations.** Skin DC were identified by flow cytometry after gating for CD45+, CD11c+ and MHC-class-II+ cells (I-Ab+). Subsequently, Langerhans cells (LC) and dermal dendritic cell subsets (DDC) were dissected based on CD207 (langerin), CD103 and CD11b expression. LC were identified as CD207^+^, CD103^-^ cells. DDC were dissected into CD207^+^ CD103^+^, CD207^-^ CD11b^-^ and CD207^-^ CD11b^+^ subsets. Representative gating of n>10 experiments.
(TIF)

Figure S5 **Lung histopathology of d1 animals.** Lung sections of d1 mice exposed to imiquimod or placebo. Mean ± SEM; n ≥3.
(TIF)
