# Supplementary figures and images for: Skin TLR7 Triggering Promotes Accumulation of Respiratory Dendritic Cells and Natural Killer Cells
Source: PLoS One. 2012 Aug 22;7(8):e43320. doi: 10.1371/journal.pone.0043320 (PMC3425551; doi:10.1371/journal.pone.0043320)

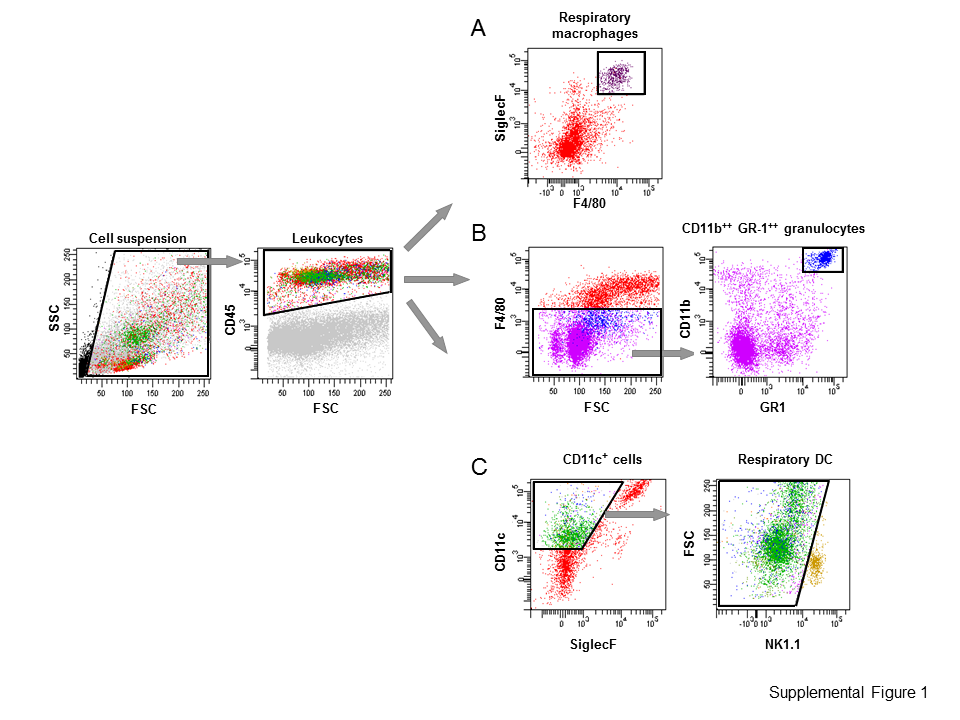

Supplement: Figure S1 — Flow-cytometry based identification of respiratory macrophages, granulocytes and DC (A-C). Leukocytes in lung cell suspensions were identified as CD45+ cells. Among the CD45+ respiratory leukocytes, macrophages were identified based on Siglec-F and F4/80 expression (A). Respiratory granulocytes were identified as CD45+ cells, F4/80low-neg and CD11b++ GR-1++ (B). Respiratory DC were identified within the CD45+ cells after gating first for the CD11c+ Siglec-Fneg cells to exclude highly autofluorescent alveolar macrophages. Subsequently, among the remaining cells, NK1.1++ NK cells were gated out to precisely identify respiratory DC (C). Representative gating of n>10 experiments. (TIF) [file pone.0043320.s001.tif]

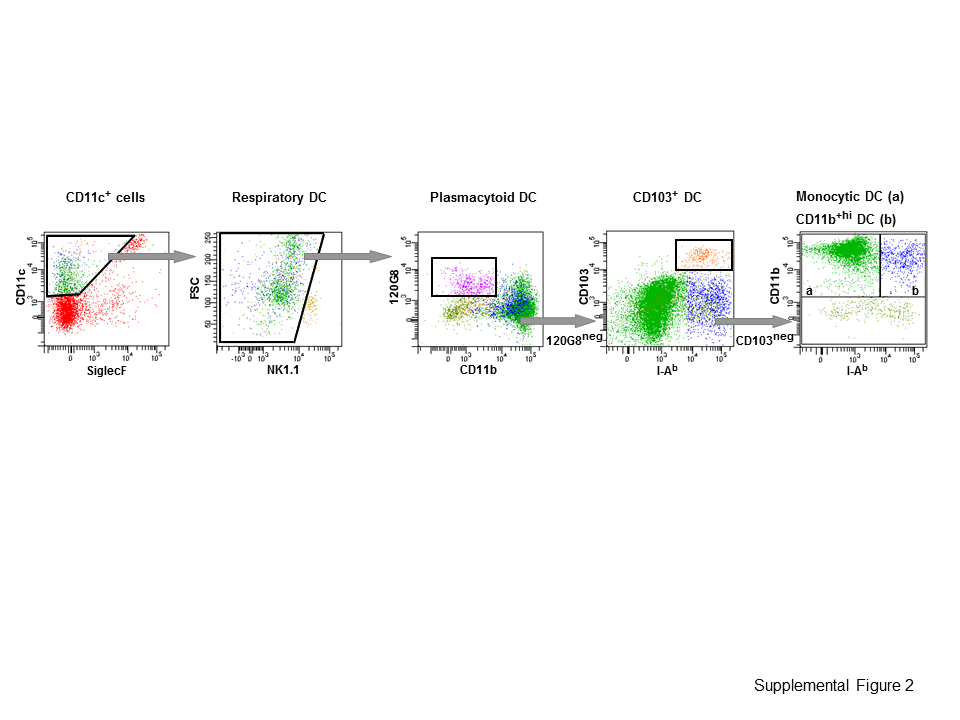

Supplement: Figure S2 — Gating strategy for discrimination of respiratory DC subsets. Respiratory DC were further dissected based on 120g8; CD103, CD11b and I-Ab (MHC-class II) expression. Among the respiratory DC fraction, plasmacytoid DC were identified based on 120g8+ CD11bneg expression. Out of the non-plasmacytoid DC fraction; CD103+ DC were identified based on CD103 and MHC-class II expression. The remaining respiratory DC were examined for CD11b and MHC-class II (I-Ab) expression dividing them into two major groups: monocytic DC (CD11b++, MHC-class IIlow, CD103neg, 120g8neg NK1.1low, Siglec Fneg, CD11c+; CD45+) and CD11bhi DC (CD11b++, MHC-IIhigh, CD103neg, 120g8neg NK1.1low, Siglec Fneg, CD11c+; CD45+). Representative gating of n>10 experiments. (TIF) [file pone.0043320.s002.tif]

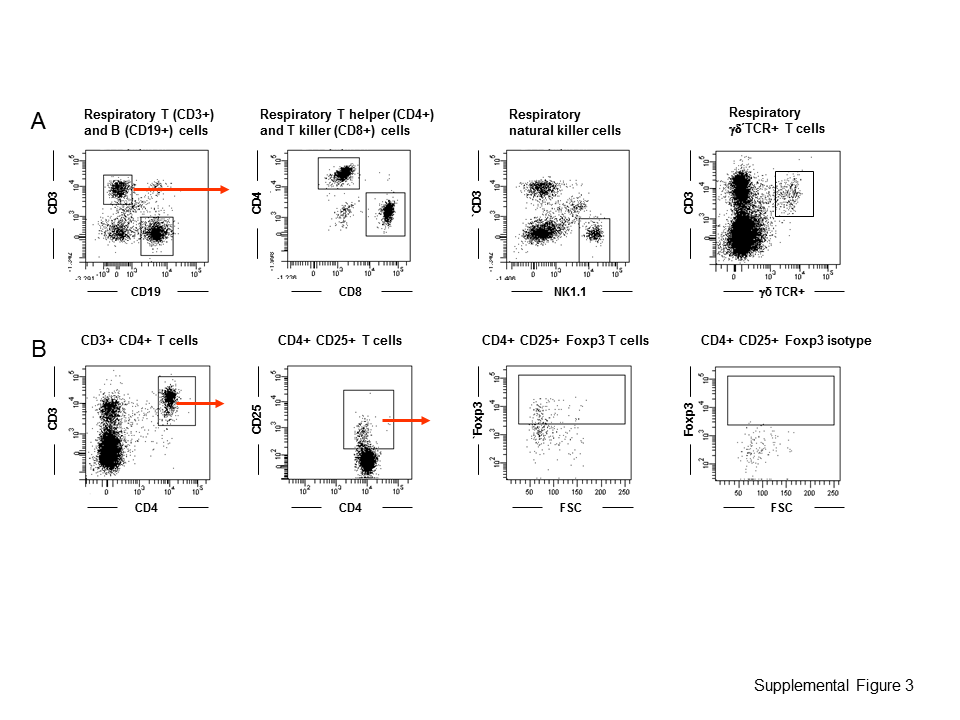

Supplement: Figure S3 — Gating strategy for discrimination of respiratory lymphocyte subpopulations (A) and regulatory T cells (B). First, classical CD3+ T cells and CD19+ B cells were identified within the CD45+ SSClow respiratory leukocytes, Out of the CD3+ T cell fraction, respiratory T helper and T killer cells were identified based on CD4 and CD8 expression, respectively (A). Additionally, among the CD45+ SSClow fraction, respiratory NK cells were identified as CD3neg NK1.1+ cells and γδ T cells were identified as CD3+ γδ TCR+ cells (A). Respiratory T regulatory cells were identified within the CD45+ SSClow fraction as CD4+CD25+ cells and then examined for Foxp3 expression (B). Foxp3 gates were set according to control staining with identical mabs except for Foxp3 isotype-matched control (so-called fluorescence minus 1 control; B). Representative gating of n>10 experiments. (TIF) [file pone.0043320.s003.tif]

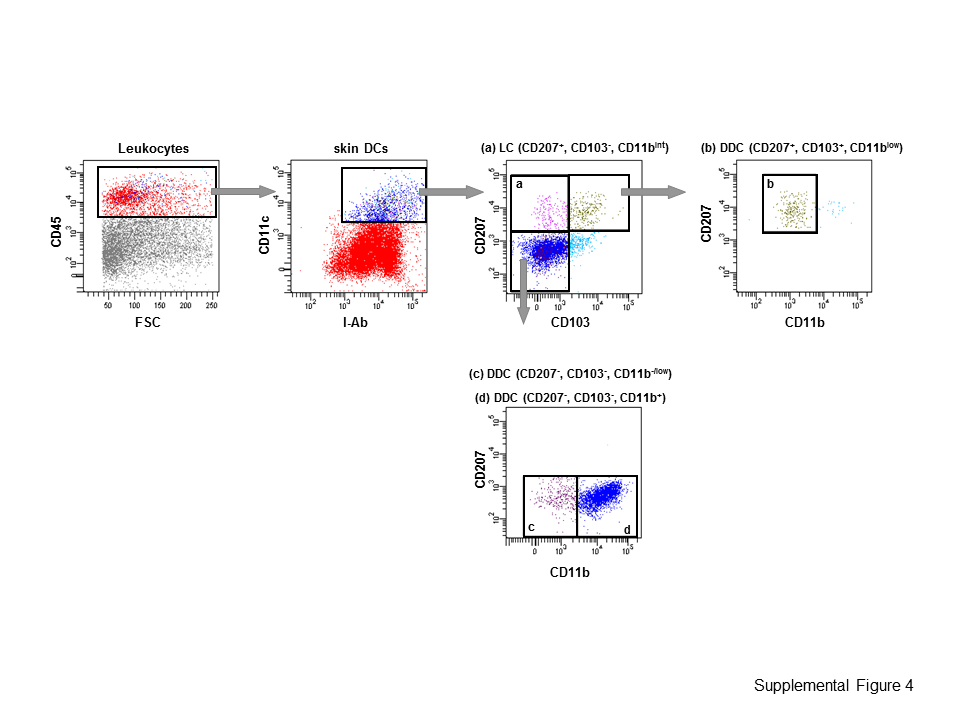

Supplement: Figure S4 — Gating strategy for discrimination of skin DC subpopulations. Skin DC were identified by flow cytometry after gating for CD45+, CD11c+ and MHC-class-II+ cells (I-Ab+). Subsequently, Langerhans cells (LC) and dermal dendritic cell subsets (DDC) were dissected based on CD207 (langerin), CD103 and CD11b expression. LC were identified as CD207+, CD103- cells. DDC were dissected into CD207+ CD103+, CD207- CD11b- and CD207- CD11b+ subsets. Representative gating of n>10 experiments. (TIF) [file pone.0043320.s004.tif]

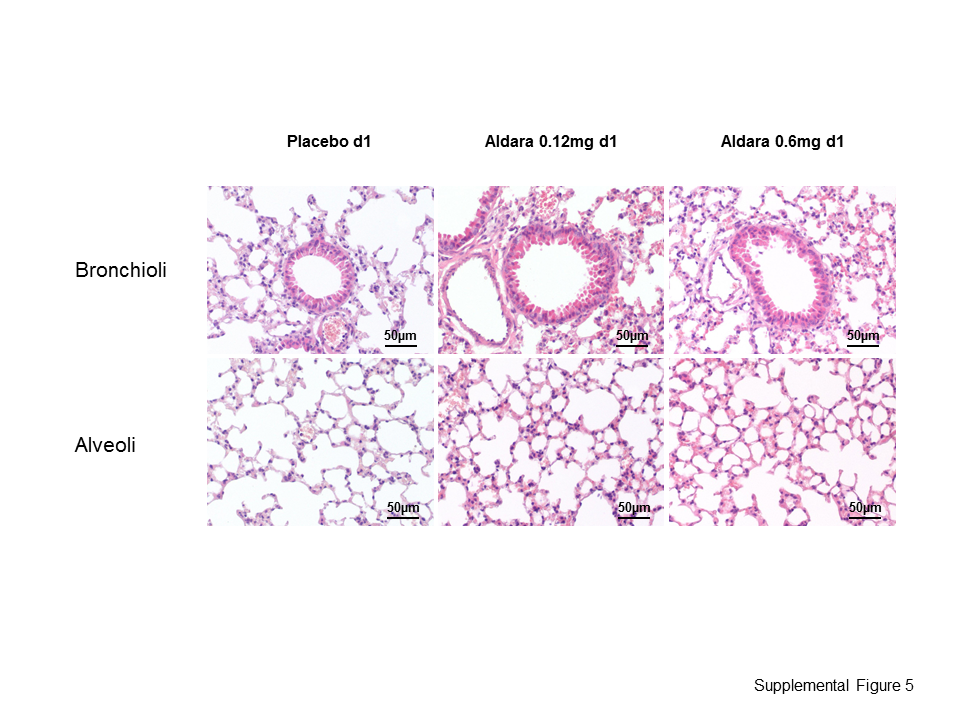

Supplement: Figure S5 — Lung histopathology of d1 animals. Lung sections of d1 mice exposed to imiquimod or placebo. Mean ± SEM; n ≥3. (TIF) [file pone.0043320.s005.tif]
